# Prospective Case Study on Characterization of Colorectal Adenomas Comparing AFI with NBI

**DOI:** 10.1155/2011/963618

**Published:** 2011-05-29

**Authors:** Haruhisa Suzuki, Yutaka Saito, Takahisa Matsuda, Takeshi Nakajima, Tsuyoshi Kikuchi

**Affiliations:** Endoscopy Division, National Cancer Center Hospital, 5-1-1 Tsukiji, Chuo-ku, Tokyo 104-0045, Japan

## Abstract

*Aim*. Compare the characterization ability of AFI and NBI for colorectal adenomas. *Methods*. We prospectively enrolled 58 patients with 89 colorectal adenomas detected by white light colonoscopy. Such lesions were subsequently observed with both AFI and NBI and then treated by endoscopic resection. With respect to the 89 lesions, 3 experienced endoscopists retrospectively evaluated the visualization quality of the AFI and NBI images in a blind manner using a three-tier scale based on excellent, fair, and poor criteria. *Results*. There were 54, 31, and 4 lesions considered as excellent, fair, and poor visualization, respectively, using AFI in comparison to 53, 19, and 17 lesions, respectively, with NBI. The percentage of excellent and fair visualization lesions was 95.5% with AFI and 80.9% with NBI (*P* < .01). *Conclusion*. This study indicated that AFI may be more effective for the characterization of colorectal adenomas because of better visualization of such lesions compared to NBI.

## 1. Introduction

Colorectal cancer is the fourth most common form of cancer and the second leading cause of cancer-related deaths in the United States [1]. Current trends suggest that colorectal cancer will soon become a major cause of morbidity and mortality in Japan as well [2] so detection and removal of colorectal adenomas by colonoscopy is becoming an increasingly important means of preventing such cancer [3].

Small or flat adenomas may be missed, however, during conventional colonoscopy examinations [4, 5]. In particular, depressed type colorectal tumors and nongranular type laterally spreading tumors (LST-NGs) have a high potential for malignancy [6, 7] even those smaller in size, but such lesions can be difficult to detect using standard white light colonoscopy (WLC). Although chromoendoscopy provides advantages over conventional colonoscopy in the detection of small lesions, the procedure is more complicated and takes longer [8]. In order to detect colorectal adenomas without the necessity of using chromoendoscopy, therefore, a need exists for the development of a new effective endoscopic method for that specific purpose.

The autofluorescence imaging (AFI) [9–12] and narrow-band imaging (NBI) [13–16] videoendoscope systems are recently developed noninvasive optical-digital imaging processes. It has been reported that both systems have an advantage over standard WLC in terms of providing better visualization and, therefore, may be able to improve the endoscopic characterization of colorectal adenomas. There have been no published reports, however, that have actually compared the characterization ability of AFI and NBI for colorectal adenomas based on the visualization of such lesions so we decided to conduct such a study.

## 2. Materials and Methods

### 2.1. Endoscopic Imaging Systems : AFI Videoendoscope System and NBI Videoendoscope System

The AFI videoendoscope system (Olympus Medical Systems Corp., Tokyo, Japan) is a new illumination method that allows for real-time white light endoscopy [9–12]. Neoplastic areas involve a thickening of the mucosal layer and increased hemoglobin so such areas emit weaker autofluorescence compared to nonneoplastic areas. Recently, the AFI system has been used to enhance detection of early lesions in the esophagus, stomach, and colon.

The NBI system (Olympus Medical Systems Corp.) is another novel optical-digital imaging process that uses special narrow-band filters in the endoscopic system to provide a more detailed visualization of the mucosal architecture and capillary pattern [13–16]. As a result of the improved mucosal contrast provided by NBI, this technique also has the potential for improving the detection of colorectal lesions compared to standard WLC.

The prototype colonoscope used for AFI and NBI examinations in this study had a sequential green and blue light source (XCLV-260HP) and a high-resolution videoendoscope (XCF-H240FZI) and video system (XCV-260HP). This endoscope also had two sets of charge-coupled devices; one for conventional white light imaging and NBI and the other for AFI. The endoscope's light source consisted of three types: conventional white light; AFI light comprised of a blue light for emitting and a green light for hemoglobin absorption; NBI light of two wavelengths for hemoglobin absorption. During the endoscopy procedures, the colonoscopist could switch from conventional imaging to AFI or NBI merely by pushing a button on the control handle of the endoscope. In addition, the videoendoscope was equipped with an accessory channel having an internal diameter of 3.2 mm. The outer diameter of the distal tip of the videoendoscope was 14.8 mm and the videoendoscope included functions for variable stiffness and magnification up to 75x with the white light image.

### 2.2. Patients

From June 2006 to October 2006, a total of 58 consecutive patients (males/females, 40/18; mean age, 63.7 ± 7.7 years, range 44–75 years) underwent colonoscopies during which a total of 89 colorectal adenomas were detected by high-resolution WLC and subsequently examined using both AFI and NBI. All lesions were treated by endoscopic resection and included in this prospective study at the National Cancer Center Hospital in Tokyo. The 89 colorectal adenomas were classified according to histology: low-grade dysplasia/high-grade dysplasia, 79/10; macroscopic type: IIa/Is, 68/21; tumor size: ≦5 mm/>5 mm, 66/23 (Table 1). Eligible patients were adults with no history of surgical resection of the colon or rectum and without inflammatory bowel disease or familial adenomatous polyposis (FAP). Written informed consent was obtained from all patients before their examinations.

### 2.3. Endoscopic Examinations

Patients prepared for their colonoscopy examinations by ingesting 2-3 liters of polyethylene glycol-electrolyte solution in the morning. Every procedure was performed by one highly experienced colonoscopist (TM) in our endoscopy division.

First, routine endoscopic examinations were performed using the white light mode of the AFI videoendoscope system to identify lesions suspected of being colorectal adenomas. If such a colorectal lesion was detected, the colonoscopist conducted AFI and NBI examinations by switching first to the AFI mode followed by the NBI mode. Photographs depicting the colorectal lesion in the center of the endoscopic monitor were then taken of the AFI and NBI views. In addition, chromoendoscopy was performed to diagnose the detected lesion more precisely.

### 2.4. Endoscopic Images of Colorectal Adenomas

A lesion suspected of being a colorectal adenoma using AFI was defined as a purple or magenta demarcated area on a green background while a lesion suspected of being a colorectal adenoma using NBI was defined as a demarcated area brownish in color.

### 2.5. Histological Assessment

We subsequently performed endoscopic resections for all visualized lesions diagnosed as being colorectal adenomas and histological examinations were conducted on all resected specimens according to World Health Organization criteria [17].

### 2.6. Evaluation of Colorectal Adenoma Visualization

During the endoscopic examinations referred to above, the endoscopist took pictures of abnormal mucosal areas and a representative collection was then assembled of both AFI and NBI images of each colorectal adenoma. After the endoscopic examinations, three other endoscopists with extensive experience in the diagnosis of colorectal adenomas (HS, TK, and YS) evaluated those lesions histologically diagnosed as being colorectal adenomas in terms of the visualization quality of the AFI and NBI images that were randomly displayed without reference to any information concerning the nature of the lesions.

The visualizations were evaluated on a three-tier scale: excellent, fair, and poor. An “excellent visualization” was defined as a lesion that could be clearly described by AFI or NBI and definitely diagnosed endoscopically as a colorectal adenoma. A “fair visualization” was defined as a lesion that could be reasonably described by AFI or NBI and diagnosed endoscopically as a colorectal adenoma although a part of the lesion's margin appeared dim. A “poor visualization” was defined as a lesion that could not be clearly described by AFI or NBI and could barely be diagnosed endoscopically as a colorectal adenoma.

Next, we confirmed the visualization scales of each lesion that had been agreed on by at least two endoscopists. In addition, we calculated the percentage of excellent and fair visualized lesions using AFI and NBI, respectively, and then compared the visualization results for the AFI and NBI images. Interobserver agreement was also assessed in relation to the visualization of colorectal adenomas.

### 2.7. Statistical Analysis

McNemar's Test was used for statistical analysis with the standard computer software statistical package SPSS for Windows (SPSS, Release 6.0; SPSS Inc., Chicago, Ill, USA, 1993) with a *P*-value <.05 considered significant. Interobserver agreement was calculated using kappa (*κ*) statistics.

## 3. Results

**Figure 1:**
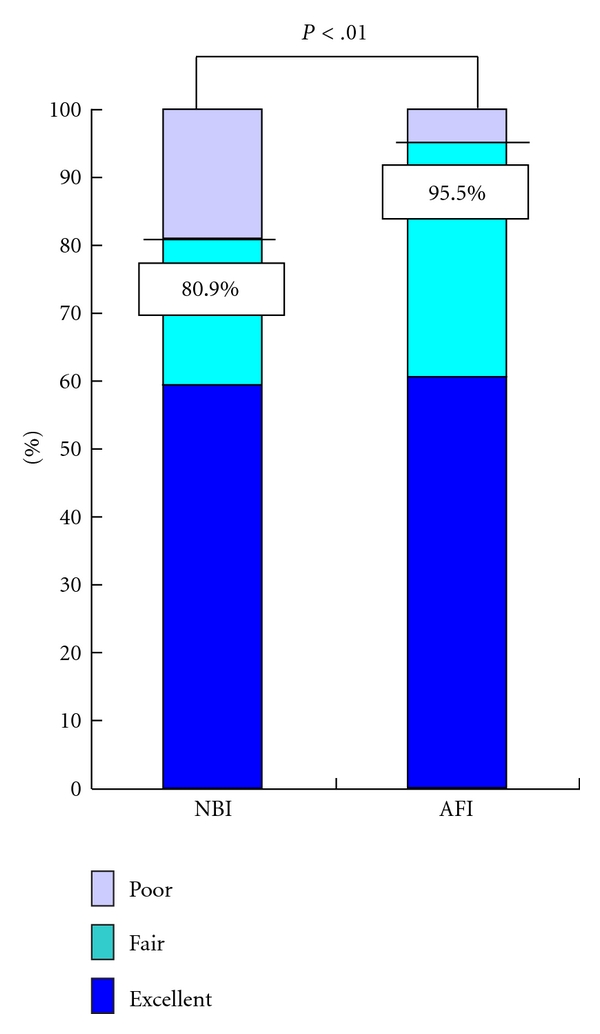
Visualization of colorectal adenomas by AFI and NBI. The percentage of lesions visualized as being excellent and fair was 95.5% with AFI and 80.9% with NBI (*P* < .01). The *P*-value was calculated using McNemar's Test.

A total of 54, 31, and four such lesions were evaluated as having excellent, fair, and poor visualization, respectively, using AFI in comparison to 53, 19, and 17 such lesions, respectively, with NBI (Table 2). Significantly more colorectal adenomas could be described by AFI compared to NBI as the percentage of excellent and fair visualized lesions was 95.5% with AFI and 80.9% with NBI (*P* < .01) (Figure 1). As for Interobserver agreement in the visualization of colorectal adenomas, there was better agreement with AFI (*κ* = 0.41) than with NBI (*κ* = 0.32).

**Figure 2:**
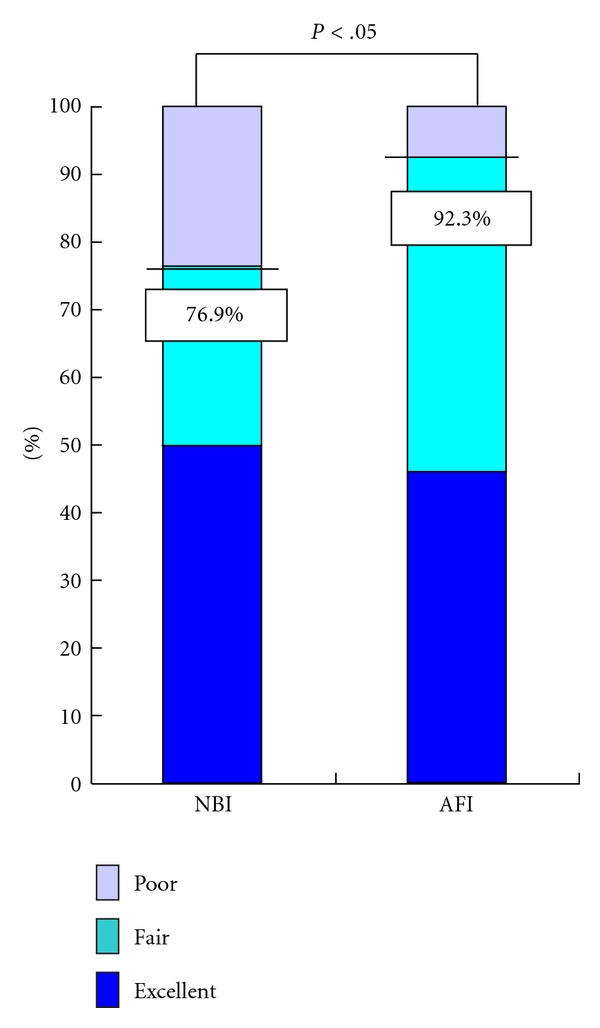
Visualization of colorectal adenoma flat lesions ≦5 mm in size by AFI and NBI. The percentage of lesions visualized as being excellent and fair was 92.3% with AFI and 76.9% with NBI (*P* < .05). The *P*-value was calculated using McNemar's Test.

With respect to 52 flat lesions (IIa) ≦5 mm in size, there were 24, 24, and four such lesions evaluated as having excellent, fair, and poor visualization, respectively, with AFI compared to 26, 14, and 12 such lesions, respectively, with NBI (Table 3). Significantly more colorectal adenomas consisting of flat lesions ≦5 mm in size could also be described by AFI compared to NBI as the percentage of excellent and fair visualized lesions was 92.3% with AFI and 76.9% with NBI (*P* < .05) (Figure 2).

**Figure 3:**
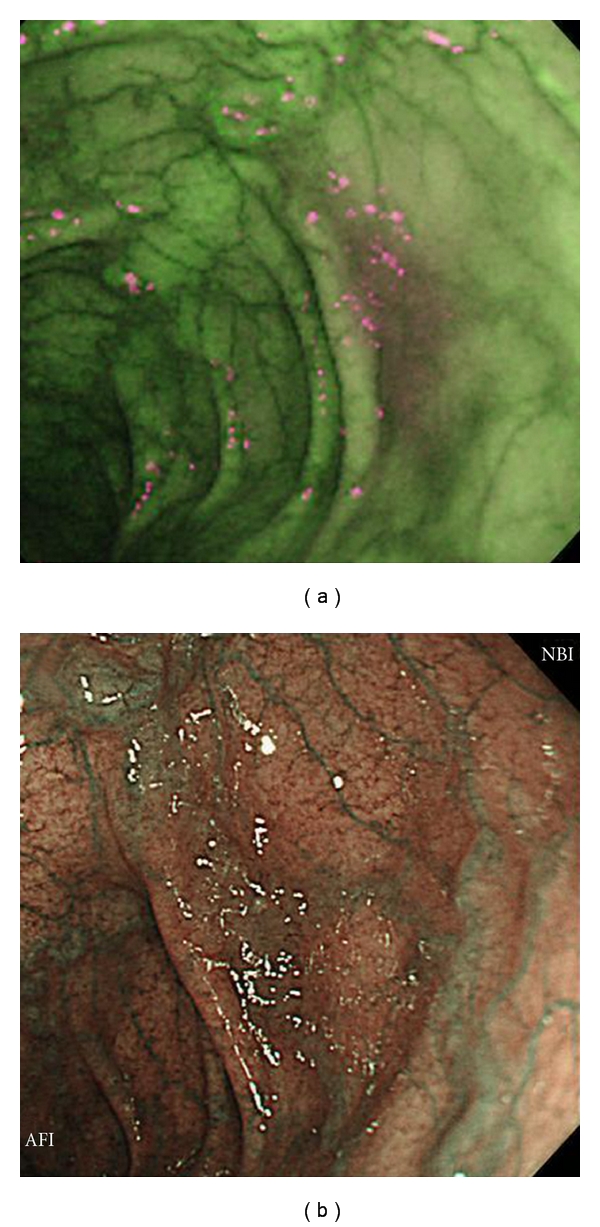
Adenoma in transverse colon (IIa, 12 mm, high-grade dysplasia). (a) A clearly demarcated area magenta in color was evaluated as being an excellent visualization by AFI. (b) NBI was unable to clearly describe this lesion resulting in a poor visualization evaluation.

**Figure 4:**
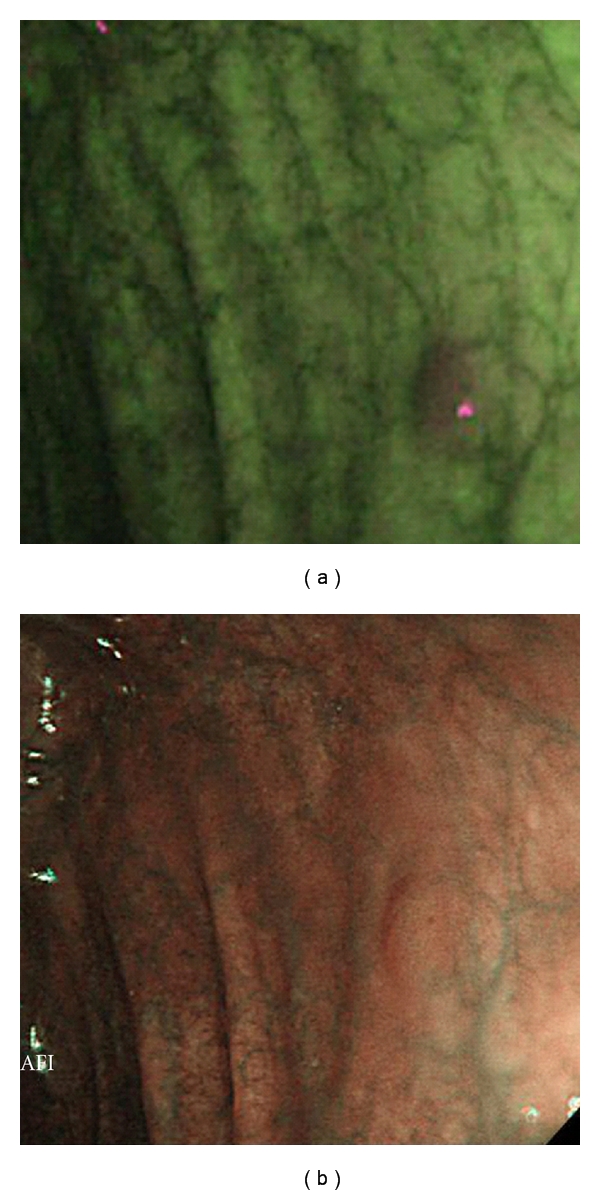
Adenoma in transverse colon (IIa, 3 mm, low-grade dysplasia). (a) A clearly demarcated area magenta in color was evaluated as being an excellent visualization by AFI. (b) NBI was unable to clearly describe this lesion resulting in a poor visualization evaluation.

AFI and corresponding NBI images of two representative adenomas both located in the transverse colon are shown in Figures 3(a) and 3(b) and Figures 4(a) and 4(b), respectively.

## 4. Discussion

Based on the results of our study, the AFI videoendoscope system demonstrated significantly better visualization of colorectal adenomas compared to the NBI system. These results suggest, therefore, that AFI may be more effective for the characterization of colorectal adenomas than NBI. In addition, AFI was able to visualize colorectal adenomas consisting of flat and smaller lesions significantly better than NBI indicating that AFI may be more effective in improving the characterization of depressed-type tumors and LST-NGs both of which have a high potential for malignancy, but are particularly difficult to visualize using conventional WLC [6, 7].

Colorectal cancer is fast becoming a major cause of cancer-related deaths in Japan so the detection of colorectal adenomas by colonoscopy is increasingly important because of the well-established connection of such lesions with colorectal cancer [2, 3] although 17–24% of colorectal adenomas are missed during conventional colonoscopy [4, 5]. Although chromoendoscopy improves the detection of small and flat colorectal adenomas compared to conventional WLC, this procedure requires considerable time for dye-spraying and observation [8]. Consequently, the development of a new, noninvasive diagnostic modality is highly desirable for the detection of colorectal adenomas.

The AFI [9–12] and NBI [13–16] videoendoscope systems could each play an important role in the future detection of colorectal adenomas because both systems have been shown to improve the endoscopic visualization of colorectal adenomas compared to WLC. Although the value of these systems has been recognized in a number of studies, there have been no published reports as yet actually comparing the characterization of colorectal adenomas using AFI and NBI. Accordingly, our research findings indicating that AFI may be of greater potential use in characterizing colorectal adenomas compared to NBI is especially important.

The NBI system [13–16] is a novel and noninvasive optical-digital imaging method that uses reflected light to visualize the superficial structure of tissue surfaces. It has been reported that NBI colonoscopy improves the detection of colorectal neoplasias [16]. Rastogi et al. also reported that NBI can lead to the detection of additional colorectal polyps missed by WLC because of the increased contrast between polyps and surrounding mucosa with NBI [18]. In addition, NBI with magnification has the potential for differentiating hyperplastic from adenomatous polyps because it can reveal surface mucosal and vascular patterns [13–15, 18]. Two other studies reported, however, that a “WLC followed by NBI” protocol cannot be recommended for colorectal cancer screening because NBI did not detect more adenomas than conventional WLC [18, 19]. Recent reports [16, 19, 20] comparing NBI with WLC, therefore, have shown conflicting results.

The AFI videoendoscope system, meanwhile, can distinguish neoplastic from nonneoplastic tissue based on differences in the intensity of the autofluorescence and green reflectance spectra [9–12]. The feasibility of using AFI for detecting cancers in the digestive tract including the esophagus, stomach, and colon has been reported as has the effectiveness of the AFI system for the detection of colorectal neoplasias [9, 11]. In addition, McCallum et al. reported that AFI colonoscopy may be a valuable tool for the virtual distinction between adenomatous and hyperplastic polyps [12].

In the present study, AFI provided superior visualization of colorectal adenomas compared to NBI so it seems reasonable to conclude from our findings that the AFI system can improve the accurate characterization of colorectal adenomas compared to NBI because of the enhanced endoscopic visualization capability of AFI. It should be noted, however, that this study was not a comparison of AFI and NBI in the detection of colorectal adenomas, but rather a trial study comparing AFI to NBI for the characterization of such lesions previously detected by WLC. In addition, there was better Interobserver agreement in the visualization of colorectal adenomas with AFI (*κ* = 0.41) than NBI (*κ* = 0.32), but the *κ*-value was low for both methods despite such variability being assessed among three experienced endoscopists. The reason for such low variability at that time could have been that a difference in the recognition and characterization of colorectal adenomas using these two new image-enhanced endoscopy diagnostic modalities existed among even experienced endoscopists although a lesion suspected of being a colorectal adenoma using each modality was basically defined as a demarcated area with a specific color. In time, such low variability should have improved, therefore, by reducing the difference in the recognition and characterization of colorectal adenomas using AFI and NBI so that a randomized controlled trial with back-to-back blind colonoscopy can be conducted in the future to compare the colorectal adenoma characterization and detection ability of not only AFI and NBI, but also WLC.

In conclusion, the results of this study indicated that the AFI videoendoscope system may be more effective for the characterization of colorectal adenomas because of better visualization of such lesions compared to the NBI videoendoscope system.

##  Conflict of Interests

All authors have no conflict of interests or financial ties to disclose.

## Figures and Tables

**Table 1:** Clinicopathological features of 89 colorectal adenomas.

Clinicopathological features	Number of lesions
Histology	
Low-grade dysplasia	79
High-grade dysplasia	10

Macroscopic type	
IIa	68
Is	21

Lesion size	
≦5 mm	66
>5 mm	23

Total	89

**Table 2:** Visualization of 89 colorectal adenomas by AFI and NBI.

	Excellent or fair visualization lesions by AFI	Poor visualization lesions by AFI	Total
Excellent or fair visualization lesions by NBI	70	2	72
Poor visualization lesions by NBI	15	2	17

Total	85	4	89

Abbreviations: AFI: Autofluorescence Imaging; NBI: Narrow-Band Imaging.

**Table 3:** Visualization of 52 colorectal adenoma flat lesions ≦5 mm in size by AFI and NBI.

	Excellent or fair visualization lesions by AFI	Poor visualization lesions by AFI	Total
Excellent or fair visualization lesions by NBI	38	2	40
Poor visualization lesions by NBI	10	2	12

Total	48	4	52

Abbreviations: AFI: Autofluorescence Imaging; NBI: Narrow-Band Imaging.
